# Ethyl 1,4-bis­(4-chloro­phen­yl)-2-methyl-1*H*-pyrrole-3-carboxyl­ate

**DOI:** 10.1107/S1600536813019247

**Published:** 2013-07-17

**Authors:** K. N. Nandeesh, Chandra, M. Mahendra, K. Palani, K. Mantelingu

**Affiliations:** a Department of Studies in Chemistry, Manasagangotri, University of Mysore, Mysore 570 006, India; b Department of Studies in Physics, Manasagangotri, University of Mysore, Mysore 570 006, India; c SER-CAT, APS, Argonne National Laboratory, Argonne, IL 60439, USA

## Abstract

In the title mol­ecule, C_20_H_17_Cl_2_NO_2_, the pyrrole moiety makes dihedral angles of 63.42 (11) and 70.43 (12)° with the chlorobenzene rings. The eth­oxy­carbonyl unit is present in a synperiplanar conformation with respect to the pyrrole ring, as indicated by the dihedral angle of 14.5 (3)°. In the crystal, mol­ecules are linked into chains parallel to the *a*-axis direction by weak C—H⋯O hydrogen bonds.

## Related literature

For the biological importance of pyrroles, see: Banwell *et al.* (2006 ▶); Mohamed *et al.* (2009 ▶); Sosa *et al.* (2002 ▶).
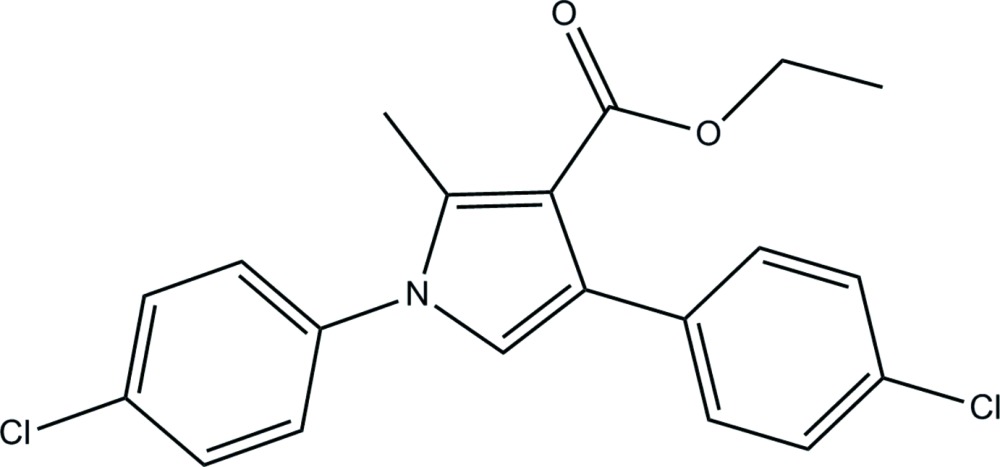


## Experimental

### 

#### Crystal data

C_20_H_17_Cl_2_NO_2_
*M* *_r_* = 374.25
Triclinic, 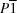

*a* = 8.037 (2) Å
*b* = 9.797 (3) Å
*c* = 12.510 (4) Å
α = 72.774 (16)°
β = 86.838 (16)°
γ = 76.804 (16)°
*V* = 915.9 (5) Å^3^
*Z* = 2
Mo *K*α radiation
μ = 0.37 mm^−1^
*T* = 296 K
0.15 × 0.15 × 0.15 mm

#### Data collection

Bruker SMART APEXII CCD area-detector diffractometer
Absorption correction: multi-scan (*SADABS*; Sheldrick, 2001 ▶) *T*_min_ = 0.947, *T*_max_ = 0.947
15843 measured reflections
4196 independent reflections
2759 reflections with *I* > 2σ(*I*)
*R*_int_ = 0.032

#### Refinement

*R*[*F* ^2^ > 2σ(*F* ^2^)] = 0.044
*wR*(*F* ^2^) = 0.125
*S* = 1.03
4196 reflections
228 parameters
H-atom parameters constrained
Δρ_max_ = 0.24 e Å^−3^
Δρ_min_ = −0.29 e Å^−3^

### 

Data collection: *APEX2* (Bruker, 2009 ▶); cell refinement: *SAINT* (Bruker, 2009 ▶); data reduction: *SAINT*; program(s) used to solve structure: *SHELXS97* (Sheldrick, 2008 ▶); program(s) used to refine structure: *SHELXL97* (Sheldrick, 2008 ▶); molecular graphics: *PLATON* (Spek, 2009 ▶); software used to prepare material for publication: *SHELXL97*

## Supplementary Material

Crystal structure: contains datablock(s) global, I. DOI: 10.1107/S1600536813019247/fb2292sup1.cif

Structure factors: contains datablock(s) I. DOI: 10.1107/S1600536813019247/fb2292Isup2.hkl

Supplementary material file. DOI: 10.1107/S1600536813019247/fb2292Isup3.cml

Additional supplementary materials:  crystallographic information; 3D view; checkCIF report

## Figures and Tables

**Table 1:** Hydrogen-bond geometry (Å, °)

*D*—H⋯*A*	*D*—H	H⋯*A*	*D*⋯*A*	*D*—H⋯*A*
C2—H2⋯O8^i^	0.93	2.58	3.453 (3)	157
C6—H6*C*⋯O8	0.96	2.42	3.041 (3)	122

Symmetry code: (i) 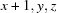.

**Fig. 1.**
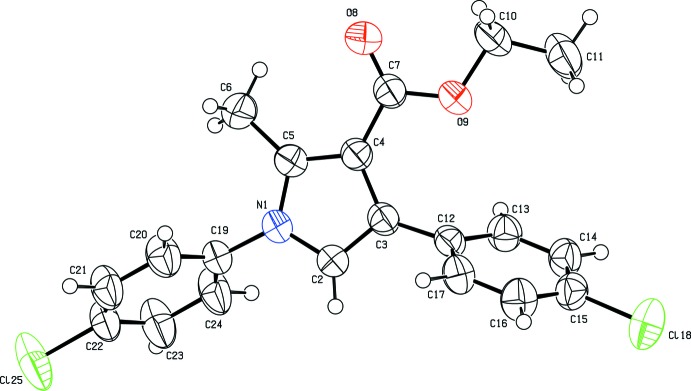
The title molecule with the labelling scheme. The displacement ellipsoids are shown at the 50% probability level.

**Fig. 2.**
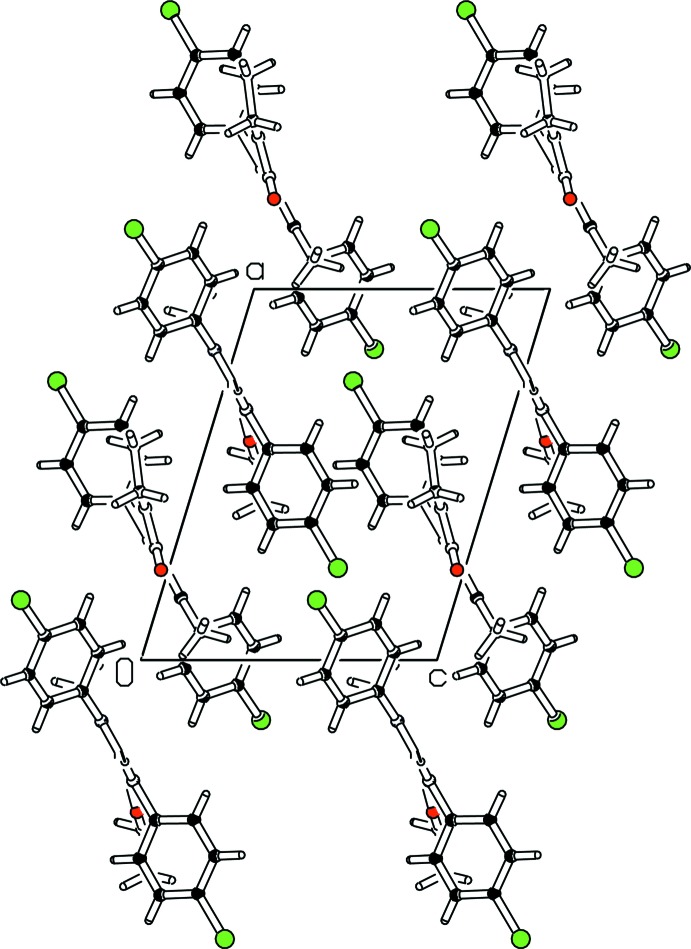
Packing diagram of the molecule viewed parallel to the a axis.

## supplementary crystallographic information

### Comment

Pyrrole is a five-membered heterocyclic ring with one nitrogen atom. Its derivatives exhibit a variety of biological activities such as antitumor (Banwell *et al.*, 2006) and antimicrobial (Mohamed *et al.*, 2009) activities. They also inhibit protein kinase (Sosa *et al.*, 2002). With this background of pyrrole derivatives, we have synthesized the title compound in order to study its crystal structure.

In the molecular structure of the title compound (Fig. 1), the dihedral angle between the pyrrole ring (N1/C2/C3/C4/C5) with phenyl rings (C19/C20/C21/C22/C23/C24) and (C12/C13/C14/C15/C16/C17) are 63.42 (11)° and 70.43 (12)°, respectively. The ethoxycarbonyl unit is in *syn-periplanar* conformation with respect to the pyrrole moiety, as indicated by the dihedral angle value of 14.5 (3)° (C3/C4/C7/O9). There are no classical hydrogen bonds and the crystal structure is stabilized by C—H···O hydrogen bonds only (see Table 1). C6—H6C···O8 forms an intramolecular hydrogen bond, while C2—H2···O8 links molecules which are parallel to the axis *a*. The packing of the molecules is shown in Fig. 2.

### Experimental

To a stirred solution of *para*-chloroaniline (1.5 mmol), *para*-chlorobenzaldehyde (1.0 mmol) and ethyl acetoacetate (1.0 mmol) in nitromethane (1.5 ml), ferric chloride (FeCl_3_) (0.1 mmol) was added. The mixture was refluxed at 90–100°C for 6 hrs and then cooled to room temperature. The excess of solvent was removed under vacuum and the residue was directly purified by column chromatography using 60–120 silica gel with ethyl acetate in hexane (1:9) as eluent which afforded the desired product as yellow solid with 88% yield. The crude product has been recrystallized from hot ethanol. Typical size of the block-shaped crystals was 0.20 × 0.15 × 0.10 mm.

### Refinement

All the H atoms were located in the difference electron density map. Nevertheless all the H atoms were situated into the idealized positions and allowed to ride on their parent atoms with C–H distances equal to 0.93, 0.96 and 0.97Å for aryl, methylene and methyl hydrogens. *U*_iso_H_aryl/methylene_ = 1.2*U*_eq_C_aryl/methylene_ and *U*_methyl_ = 1.5*U*_eq_C_methyl_

### Crystal data

**Table:**

C_20_H_17_Cl_2_NO_2_	*Z* = 2
*M**_r_* = 374.25	*F*(000) = 388
Triclinic, *P*1	*D*_x_ = 1.357 Mg m^−^^3^
Hall symbol: -P 1	Mo *K*α radiation, λ = 0.71073 Å
*a* = 8.037 (2) Å	Cell parameters from 4196 reflections
*b* = 9.797 (3) Å	θ = 1.7–27.5°
*c* = 12.510 (4) Å	µ = 0.37 mm^−^^1^
α = 72.774 (16)°	*T* = 296 K
β = 86.838 (16)°	Block, yellow
γ = 76.804 (16)°	0.15 × 0.15 × 0.15 mm
*V* = 915.9 (5) Å^3^	

### Data collection

**Table:**

Bruker SMART APEXII CCD area-detector diffractometer	4196 independent reflections
Radiation source: fine-focus sealed tube	2759 reflections with *I* > 2σ(*I*)
Graphite monochromator	*R*_int_ = 0.032
ω and φ scans	θ_max_ = 27.5°, θ_min_ = 1.7°
Absorption correction: multi-scan (*SADABS*; Sheldrick, 2001)	*h* = −10→10
*T*_min_ = 0.947, *T*_max_ = 0.947	*k* = −12→12
15843 measured reflections	*l* = −16→16

### Refinement

**Table:**

Refinement on *F*^2^	Primary atom site location: structure-invariant direct methods
Least-squares matrix: full	Secondary atom site location: difference Fourier map
*R*[*F*^2^ > 2σ(*F*^2^)] = 0.044	Hydrogen site location: difference Fourier map
*wR*(*F*^2^) = 0.125	H-atom parameters constrained
*S* = 1.03	*w* = 1/[σ^2^(*F*_o_^2^) + (0.0487*P*)^2^ + 0.2827*P*] where *P* = (*F*_o_^2^ + 2*F*_c_^2^)/3
4196 reflections	(Δ/σ)_max_ < 0.001
228 parameters	Δρ_max_ = 0.24 e Å^−^^3^
0 restraints	Δρ_min_ = −0.29 e Å^−^^3^
66 constraints	

### Special details

**Table:**

Geometry. Bond distances, angles *etc*. have been calculated using the rounded fractional coordinates. All su's are estimated from the variances of the (full) variance-covariance matrix. The cell e.s.d.'s are taken into account in the estimation of distances, angles and torsion angles
Refinement. Refinement on *F*^2^ for ALL reflections except those flagged by the user for potential systematic errors. Weighted *R*-factors *wR* and all goodnesses of fit *S* are based on *F*^2^, conventional *R*-factors *R* are based on *F*, with *F* set to zero for negative *F*^2^. The observed criterion of *F*^2^ > σ(*F*^2^) is used only for calculating -*R*-factor-obs *etc*. and is not relevant to the choice of reflections for refinement. *R*-factors based on *F*^2^ are statistically about twice as large as those based on *F*, and *R*-factors based on ALL data will be even larger.

### Fractional atomic coordinates and isotropic or equivalent isotropic displacement parameters (Å^2^)

**Table:**

	*x*	*y*	*z*	*U*_iso_*/*U*_eq_	
Cl18	0.20768 (10)	0.57041 (7)	0.24828 (5)	0.0884 (2)	
Cl25	0.78934 (10)	−0.46743 (9)	1.16162 (6)	0.1057 (3)	
O9	−0.18070 (16)	0.14269 (16)	0.59033 (12)	0.0585 (4)	
O8	−0.27098 (18)	0.0253 (2)	0.75691 (13)	0.0735 (5)	
N1	0.2589 (2)	−0.07110 (19)	0.83555 (13)	0.0521 (4)	
C13	0.1310 (3)	0.2004 (2)	0.46496 (17)	0.0554 (5)	
H13	0.1005	0.1157	0.4616	0.067*	
C12	0.1613 (2)	0.2151 (2)	0.56891 (15)	0.0458 (4)	
C3	0.1594 (2)	0.0973 (2)	0.67518 (15)	0.0470 (4)	
C7	−0.1558 (2)	0.0648 (2)	0.69825 (16)	0.0498 (5)	
C4	0.0231 (2)	0.0321 (2)	0.73283 (15)	0.0458 (4)	
C15	0.1876 (3)	0.4336 (2)	0.37081 (17)	0.0562 (5)	
C17	0.2025 (3)	0.3436 (2)	0.56978 (17)	0.0554 (5)	
H17	0.2219	0.3576	0.6380	0.066*	
C14	0.1448 (3)	0.3078 (2)	0.36672 (17)	0.0589 (5)	
H14	0.1251	0.2949	0.2982	0.071*	
C19	0.3838 (2)	−0.1689 (2)	0.91686 (16)	0.0505 (5)	
C2	0.2999 (2)	0.0304 (2)	0.74178 (16)	0.0533 (5)	
H2	0.4079	0.0502	0.7263	0.064*	
C22	0.6316 (3)	−0.3508 (3)	1.06663 (17)	0.0615 (6)	
C16	0.2157 (3)	0.4528 (2)	0.47182 (19)	0.0640 (6)	
H16	0.2436	0.5388	0.4746	0.077*	
C6	0.0083 (3)	−0.1721 (3)	0.92294 (19)	0.0655 (6)	
H6A	−0.0078	−0.1366	0.9874	0.098*	
H6B	0.0816	−0.2684	0.9426	0.098*	
H6C	−0.1003	−0.1764	0.8973	0.098*	
C5	0.0887 (2)	−0.0707 (2)	0.83166 (16)	0.0501 (5)	
C23	0.6287 (3)	−0.3663 (3)	0.9623 (2)	0.0794 (8)	
H23	0.7100	−0.4383	0.9421	0.095*	
C20	0.3894 (3)	−0.1548 (3)	1.02199 (18)	0.0664 (6)	
H20	0.3088	−0.0826	1.0424	0.080*	
C21	0.5134 (3)	−0.2469 (3)	1.09782 (18)	0.0690 (6)	
H21	0.5162	−0.2381	1.1697	0.083*	
C24	0.5039 (3)	−0.2739 (3)	0.88682 (18)	0.0721 (7)	
H24	0.5014	−0.2832	0.8151	0.087*	
C10	−0.3529 (3)	0.1775 (3)	0.54626 (19)	0.0698 (6)	
H10A	−0.4272	0.2462	0.5798	0.084*	
H10B	−0.3972	0.0894	0.5630	0.084*	
C11	−0.3464 (3)	0.2438 (3)	0.4224 (2)	0.0822 (8)	
H11A	−0.3013	0.3303	0.4068	0.123*	
H11B	−0.4596	0.2694	0.3910	0.123*	
H11C	−0.2742	0.1744	0.3900	0.123*	

### Atomic displacement parameters (Å^2^)

**Table:**

	*U*^11^	*U*^22^	*U*^33^	*U*^12^	*U*^13^	*U*^23^
Cl18	0.1196 (6)	0.0733 (4)	0.0583 (4)	−0.0226 (4)	0.0054 (3)	0.0019 (3)
Cl25	0.1053 (6)	0.1152 (6)	0.0738 (4)	0.0282 (4)	−0.0484 (4)	−0.0242 (4)
O8	0.0469 (8)	0.1037 (13)	0.0608 (9)	−0.0179 (8)	0.0010 (7)	−0.0098 (9)
O9	0.0441 (7)	0.0719 (10)	0.0534 (8)	−0.0092 (7)	−0.0091 (6)	−0.0103 (7)
N1	0.0445 (9)	0.0604 (10)	0.0448 (9)	−0.0098 (7)	−0.0067 (7)	−0.0056 (8)
C2	0.0458 (11)	0.0623 (13)	0.0474 (11)	−0.0157 (9)	−0.0044 (8)	−0.0057 (10)
C3	0.0447 (10)	0.0518 (11)	0.0444 (10)	−0.0085 (8)	−0.0044 (8)	−0.0146 (9)
C4	0.0431 (10)	0.0495 (11)	0.0440 (10)	−0.0073 (8)	−0.0011 (8)	−0.0143 (9)
C5	0.0445 (10)	0.0551 (12)	0.0495 (11)	−0.0099 (9)	−0.0014 (8)	−0.0141 (9)
C6	0.0567 (13)	0.0673 (15)	0.0614 (14)	−0.0137 (11)	0.0008 (10)	−0.0022 (11)
C7	0.0471 (11)	0.0542 (12)	0.0470 (11)	−0.0065 (9)	−0.0031 (9)	−0.0163 (9)
C10	0.0461 (11)	0.0895 (17)	0.0670 (14)	−0.0050 (11)	−0.0159 (10)	−0.0172 (13)
C11	0.0730 (16)	0.104 (2)	0.0638 (15)	−0.0006 (14)	−0.0226 (12)	−0.0256 (14)
C12	0.0391 (9)	0.0498 (11)	0.0454 (10)	−0.0043 (8)	−0.0050 (7)	−0.0126 (9)
C13	0.0609 (12)	0.0560 (12)	0.0521 (12)	−0.0145 (10)	−0.0025 (9)	−0.0181 (10)
C14	0.0657 (13)	0.0681 (14)	0.0432 (11)	−0.0131 (11)	−0.0025 (9)	−0.0177 (10)
C15	0.0562 (12)	0.0538 (12)	0.0487 (11)	−0.0031 (9)	0.0008 (9)	−0.0072 (10)
C16	0.0803 (15)	0.0499 (12)	0.0617 (14)	−0.0140 (11)	−0.0046 (11)	−0.0157 (11)
C17	0.0637 (13)	0.0549 (13)	0.0474 (11)	−0.0092 (10)	−0.0085 (9)	−0.0161 (10)
C19	0.0464 (10)	0.0563 (12)	0.0429 (10)	−0.0089 (9)	−0.0073 (8)	−0.0059 (9)
C20	0.0676 (14)	0.0734 (15)	0.0534 (13)	0.0026 (11)	−0.0073 (10)	−0.0238 (11)
C21	0.0756 (15)	0.0849 (17)	0.0443 (11)	−0.0051 (13)	−0.0134 (10)	−0.0225 (12)
C22	0.0628 (13)	0.0675 (14)	0.0463 (11)	−0.0038 (11)	−0.0170 (9)	−0.0098 (10)
C23	0.0809 (16)	0.0853 (18)	0.0589 (14)	0.0205 (13)	−0.0208 (12)	−0.0267 (13)
C24	0.0764 (15)	0.0865 (17)	0.0474 (12)	0.0090 (13)	−0.0181 (10)	−0.0275 (12)

### Geometric parameters (Å, º)

**Table:**

Cl18—C15	1.740 (2)	C19—C24	1.366 (3)
Cl25—C22	1.739 (3)	C20—C21	1.376 (3)
O8—C7	1.211 (2)	C21—C22	1.358 (4)
O9—C7	1.340 (2)	C22—C23	1.360 (3)
O9—C10	1.447 (3)	C23—C24	1.379 (4)
N1—C2	1.375 (3)	C2—H2	0.9300
N1—C5	1.371 (2)	C6—H6A	0.9600
N1—C19	1.434 (3)	C6—H6B	0.9600
C2—C3	1.357 (3)	C6—H6C	0.9600
C3—C4	1.445 (2)	C10—H10A	0.9700
C3—C12	1.482 (3)	C10—H10B	0.9700
C4—C5	1.383 (3)	C11—H11A	0.9600
C4—C7	1.461 (2)	C11—H11B	0.9600
C5—C6	1.499 (3)	C11—H11C	0.9600
C10—C11	1.495 (3)	C13—H13	0.9300
C12—C13	1.390 (3)	C14—H14	0.9300
C12—C17	1.376 (3)	C16—H16	0.9300
C13—C14	1.379 (3)	C17—H17	0.9300
C14—C15	1.369 (3)	C20—H20	0.9300
C15—C16	1.369 (3)	C21—H21	0.9300
C16—C17	1.385 (3)	C23—H23	0.9300
C19—C20	1.367 (3)	C24—H24	0.9300
			
Cl18···C21^i^	3.505 (3)	C10···H2^v^	3.0500
Cl18···H23^ii^	3.0100	C10···H16^vii^	3.0400
Cl25···H17^iii^	3.0000	C11···H16^vii^	3.0700
O8···C6	3.041 (3)	C15···H11B^x^	2.9100
O8···C20^iv^	3.377 (3)	C17···H10A^x^	2.9100
O9···C12	2.971 (2)	C19···H6B	2.7900
O9···C13	2.957 (3)	H2···O8^x^	2.5800
O8···H10A	2.7200	H2···C10^x^	3.0500
O8···H10B	2.5300	H2···H10B^x^	2.5000
O8···H20^iv^	2.7200	H6B···C19	2.7900
O8···H21^iv^	2.8500	H6C···O8	2.4200
O8···H2^v^	2.5800	H6C···C7	2.8500
O8···H6C	2.4200	H10A···O8	2.7200
O9···H13	2.7100	H10A···C17^v^	2.9100
O9···H13^vi^	2.7300	H10B···O8	2.5300
O9···H16^vii^	2.9100	H10B···H2^v^	2.5000
C6···O8	3.041 (3)	H11A···H16^vii^	2.3500
C6···C20	3.424 (4)	H11B···C15^v^	2.9100
C12···O9	2.971 (2)	H11B···H24^vi^	2.5800
C13···O9	2.957 (3)	H11C···C2^vi^	3.0000
C15···C17^vii^	3.567 (3)	H11C···C3^vi^	2.9500
C16···C17^vii^	3.473 (3)	H13···O9	2.7100
C16···C16^vii^	3.468 (4)	H13···O9^vi^	2.7300
C17···C15^vii^	3.567 (3)	H13···C7^vi^	2.9900
C17···C16^vii^	3.473 (3)	H16···O9^vii^	2.9100
C20···O8^iv^	3.377 (3)	H16···C10^vii^	3.0400
C20···C6	3.424 (4)	H16···C11^vii^	3.0700
C21···Cl18^viii^	3.505 (3)	H16···H11A^vii^	2.3500
C23···C23^ix^	3.582 (4)	H17···C2	3.0100
C2···H11C^vi^	3.0000	H17···Cl25^iii^	3.0000
C2···H24	3.0200	H20···O8^iv^	2.7200
C2···H17	3.0100	H21···O8^iv^	2.8500
C3···H11C^vi^	2.9500	H23···Cl18^ii^	3.0100
C7···H13^vi^	2.9900	H24···C2	3.0200
C7···H6C	2.8500	H24···H11B^vi^	2.5800
			
C7—O9—C10	116.77 (15)	C19—C24—C23	120.4 (2)
C2—N1—C5	109.47 (16)	N1—C2—H2	125.00
C2—N1—C19	122.99 (16)	C3—C2—H2	125.00
C5—N1—C19	127.29 (16)	C5—C6—H6A	109.00
N1—C2—C3	109.90 (16)	C5—C6—H6B	109.00
C2—C3—C4	105.42 (16)	C5—C6—H6C	109.00
C2—C3—C12	122.85 (16)	H6A—C6—H6B	109.00
C4—C3—C12	131.70 (16)	H6A—C6—H6C	110.00
C3—C4—C5	108.37 (15)	H6B—C6—H6C	109.00
C3—C4—C7	128.27 (17)	O9—C10—H10A	110.00
C5—C4—C7	123.36 (16)	O9—C10—H10B	110.00
N1—C5—C4	106.84 (16)	C11—C10—H10A	110.00
N1—C5—C6	121.23 (18)	C11—C10—H10B	110.00
C4—C5—C6	131.92 (17)	H10A—C10—H10B	108.00
O8—C7—O9	122.15 (17)	C10—C11—H11A	109.00
O8—C7—C4	125.71 (18)	C10—C11—H11B	109.00
O9—C7—C4	112.13 (15)	C10—C11—H11C	109.00
O9—C10—C11	107.67 (19)	H11A—C11—H11B	109.00
C3—C12—C13	123.03 (18)	H11A—C11—H11C	110.00
C3—C12—C17	119.97 (17)	H11B—C11—H11C	110.00
C13—C12—C17	116.92 (18)	C12—C13—H13	119.00
C12—C13—C14	121.90 (19)	C14—C13—H13	119.00
C13—C14—C15	119.53 (19)	C13—C14—H14	120.00
Cl18—C15—C14	120.60 (16)	C15—C14—H14	120.00
Cl18—C15—C16	119.25 (17)	C15—C16—H16	120.00
C14—C15—C16	120.15 (19)	C17—C16—H16	120.00
C15—C16—C17	119.7 (2)	C12—C17—H17	119.00
C12—C17—C16	121.80 (19)	C16—C17—H17	119.00
N1—C19—C20	121.23 (19)	C19—C20—H20	120.00
N1—C19—C24	119.19 (18)	C21—C20—H20	120.00
C20—C19—C24	119.5 (2)	C20—C21—H21	120.00
C19—C20—C21	120.4 (2)	C22—C21—H21	120.00
C20—C21—C22	119.3 (2)	C22—C23—H23	120.00
Cl25—C22—C21	119.70 (17)	C24—C23—H23	120.00
Cl25—C22—C23	119.1 (2)	C19—C24—H24	120.00
C21—C22—C23	121.2 (2)	C23—C24—H24	120.00
C22—C23—C24	119.2 (3)		
			
C10—O9—C7—O8	−0.2 (3)	C7—C4—C5—C6	1.5 (4)
C10—O9—C7—C4	178.21 (19)	C3—C4—C7—O8	−167.2 (2)
C7—O9—C10—C11	−171.6 (2)	C3—C4—C7—O9	14.5 (3)
C5—N1—C2—C3	−0.5 (2)	C5—C4—C7—O8	12.2 (3)
C19—N1—C2—C3	174.18 (18)	C5—C4—C7—O9	−166.22 (19)
C2—N1—C5—C4	0.6 (2)	C3—C12—C13—C14	−175.6 (2)
C2—N1—C5—C6	179.4 (2)	C17—C12—C13—C14	1.3 (3)
C19—N1—C5—C4	−173.79 (19)	C3—C12—C17—C16	176.1 (2)
C19—N1—C5—C6	5.0 (3)	C13—C12—C17—C16	−0.9 (3)
C2—N1—C19—C20	110.8 (2)	C12—C13—C14—C15	−0.8 (4)
C2—N1—C19—C24	−66.3 (3)	C13—C14—C15—Cl18	179.48 (19)
C5—N1—C19—C20	−75.4 (3)	C13—C14—C15—C16	−0.3 (4)
C5—N1—C19—C24	107.5 (2)	Cl18—C15—C16—C17	−179.05 (19)
N1—C2—C3—C4	0.2 (2)	C14—C15—C16—C17	0.7 (4)
N1—C2—C3—C12	178.42 (18)	C15—C16—C17—C12	−0.1 (4)
C2—C3—C4—C5	0.2 (2)	N1—C19—C20—C21	−178.0 (2)
C2—C3—C4—C7	179.6 (2)	C24—C19—C20—C21	−1.0 (4)
C12—C3—C4—C5	−177.8 (2)	N1—C19—C24—C23	177.9 (2)
C12—C3—C4—C7	1.6 (4)	C20—C19—C24—C23	0.8 (4)
C2—C3—C12—C13	116.0 (2)	C19—C20—C21—C22	0.9 (4)
C2—C3—C12—C17	−60.8 (3)	C20—C21—C22—Cl25	179.8 (2)
C4—C3—C12—C13	−66.3 (3)	C20—C21—C22—C23	−0.6 (4)
C4—C3—C12—C17	116.9 (2)	Cl25—C22—C23—C24	−179.9 (2)
C3—C4—C5—N1	−0.5 (2)	C21—C22—C23—C24	0.5 (4)
C3—C4—C5—C6	−179.1 (2)	C22—C23—C24—C19	−0.6 (4)
C7—C4—C5—N1	−179.95 (18)		

Symmetry codes: (i) *x*, *y*+1, *z*−1; (ii) −*x*+1, −*y*, −*z*+1; (iii) −*x*+1, −*y*, −*z*+2; (iv) −*x*, −*y*, −*z*+2; (v) *x*−1, *y*, *z*; (vi) −*x*, −*y*, −*z*+1; (vii) −*x*, −*y*+1, −*z*+1; (viii) *x*, *y*−1, *z*+1; (ix) −*x*+1, −*y*−1, −*z*+2; (x) *x*+1, *y*, *z*.

### Hydrogen-bond geometry (Å, º)

**Table:**

*D*—H···*A*	*D*—H	H···*A*	*D*···*A*	*D*—H···*A*
C2—H2···O8^x^	0.93	2.58	3.453 (3)	157
C6—H6*C*···O8	0.96	2.42	3.041 (3)	122

Symmetry code: (x) *x*+1, *y*, *z*.

## References

[bb1] Banwell, M. G., Hamel, E., Hockless, D. C. R., Verdier-Pinard, P., Willis, A. C. & Wong, D. J. (2006). *Bioorg. Med. Chem.* **14**, 4627–4638. 10.1016/j.bmc.2006.02.018 16510287

[bb2] Bruker (2009). *APEX2* and *SAINT* Bruker AXS Inc., Madison, Wisconsin, USA.

[bb3] Mohamed, M. S., El-Domany, R. A. & El-Hameed, R. H. A. (2009). *Acta Pharm.* **59**, 145–158. 10.2478/v10007-009-0016-9 19564140

[bb4] Sheldrick, G. M. (2001). *SADABS*, University of Göttingen, Germany.

[bb5] Sheldrick, G. M. (2008). *Acta Cryst.* A**64**, 112–122. 10.1107/S0108767307043930 18156677

[bb6] Sosa, A. C. B., Yakushijin, K. & Horne, D. A. (2002). *J. Org. Chem.* **67**, 4498–4500. 10.1021/jo020063v 12076147

[bb7] Spek, A. L. (2009). *Acta Cryst.* D**65**, 148–155. 10.1107/S090744490804362X PMC2631630 19171970

